# Application of Hansen Solubility Parameters for Green Solvent Extraction of Flavonoids from *Oroxylum indicum* (L.) Kurz and Chromatographic Profiling Across Plant Organs

**DOI:** 10.3390/molecules31203586

**Published:** 2026-10-09

**Authors:** Prommin Suphanakron, Rutchaphol Sookjitt, Wullapa Jittachai, Chatchapong Tangjidapichai, Weerasak Samee

**Affiliations:** Faculty of Pharmacy, Srinakharinwirot University, Nakhon Nayok 26120, Thailand; prommin.suphanakron@g.swu.ac.th (P.S.); rutchaphol.sookjitt@g.swu.ac.th (R.S.); wullapa@g.swu.ac.th (W.J.); chatchapong@g.swu.ac.th (C.T.)

**Keywords:** extraction, green solvent, flavonoids, ultrasonic, chromatography, sustainable

## Abstract

*Oroxylum indicum* (L.) Kurz is an edible medicinal plant whose pharmacological properties are largely attributed to four flavonoids: baicalein, oroxin A, oroxin B, and chrysin. This study developed an ultrasound-assisted extraction method using environmentally benign ethanol–water mixtures selected with Hansen solubility parameters (HSPs) to recover these compounds from six plant tissues: seeds, leaves, roots, bark, pod husks, and flowers. A high-performance liquid chromatography method was developed and validated for their quantitative determination. Comparative tissue profiling showed that seeds contained the highest concentrations of all four flavonoids. Baicalein and chrysin exhibited maximum recoveries with 60% ethanol, yielding 53.47 ± 1.07 and 28.11 ± 0.68 mg/g dry weight, respectively, only baicalein values being consistent with HSP-based predictions. In contrast, oroxin B and oroxin A were extracted most efficiently with 80% ethanol, reaching 65.18 ± 1.09 and 128.38 ± 1.88 mg/g dry weight, respectively, contrary to predictions for the glycosylated flavonoids. These results demonstrate that HSPs can effectively support green-solvent selection for baicalein extraction, although their predictive performance may be limited for flavonoid glycosides and chrysin. The method’s sustainability was confirmed using AGREEprep™, which produced a score of 0.67, compared with 0.53 for a petroleum ether–methanol system. This work provides a practical foundation for sustainable extraction and standardization of *O. indicum* for pharmaceutical applications.

## 1. Introduction

*Oroxylum indicum* (L.) Kurz is a medicinal tree native to South and Southeast Asia, long employed in traditional medical systems for its anti-inflammatory, antioxidant, antimicrobial and neuroprotective activities [1,2,3,4,5]. Phytochemical studies have revealed a diverse array of secondary metabolites in *O. indicum*, in particular flavonoids such as baicalein, chrysin and oroxylin A and their glycosides (e.g., oroxin A, oroxin B), which are major contributors to the species’ bioactivity [6,7,8]. Systematic profiling of flavonoid composition across distinct plant organs—leaves, bark, roots, flowers, pod husk and seeds—is therefore critical for elucidating biosynthetic distribution and for identifying the most appropriate tissue sources for extraction and downstream applications.

Conventional flavonoid extraction typically employs volatile organic solvents (e.g., methanol, acetone, ethyl acetate, chloroform), which raise environmental, health and safety concerns and often necessitate extensive solvent removal and purification. The imperative for sustainable natural-product processing has therefore driven the development of green extraction methodologies that minimize toxicity, energy consumption and ecological impact while preserving or enhancing extraction efficiency and selectivity. Consequently, the choice of solvent system emerges as a pivotal factor influencing extraction yield, selectivity toward target analytes, and compatibility with subsequent analytical procedures [9,10,11,12].

Hansen solubility parameters (HSP) offer a quantitative framework for predicting solute–solvent affinity by partitioning intermolecular interactions into dispersive, polar and hydrogen-bonding components. Mapping solutes and candidate solvents within HSP space enables rational screening of individual solvents or mixtures likely to solubilize particular classes of phytochemicals, thereby reducing the need for exhaustive empirical testing. Applied to natural-product extraction, HSP facilitates the targeted selection of greener solvent alternatives—including bio-based solvents, deep eutectic solvents, and benign polar protic or aprotic solvents—that optimize solvency while accounting for sustainability considerations [13,14,15]. However, extraction efficiency is also influenced by matrix-related factors, including the binding of analytes to cellular components, tissue structure, solvent penetration, analyte accessibility, diffusion, and mass-transfer resistance. In addition, during ultrasound-assisted extraction, cavitation, cell disruption, and enhanced solvent penetration may substantially affect analyte release [16,17,18,19]. Consequently, HSP compatibility alone may not fully predict the actual extraction performance of a solvent, particularly for flavonoids differing in polarity and molecular structure and occurring in different plant organs. Further investigation is therefore needed to determine the extent to which HSPs can predict flavonoid extraction from complex plant matrices and to clarify their relationship with matrix effects and ultrasound-assisted mass transfer. The present study addresses this gap by evaluating HSP-guided solvent selection for the extraction of four structurally diverse flavonoids from different organs of *O. indicum*.

A water–ethanol mixture is a preferred green solvent system, combining two non-toxic, biodegradable, and renewable components. Adjusting the water:ethanol ratio enables straightforward modulation of solvent polarity to dissolve both polar and moderately nonpolar compounds, thereby reducing reliance on petroleum-derived reagents [20,21,22]. Ultrasound-assisted extraction (UAE) is widely regarded as an effective technique for disrupting plant cell walls, enhancing solvent penetration and mass transfer, and promoting release of target analytes; its performance, however, is contingent upon solvent choice [23,24,25]. Consequently, the integration of UAE with green solvent systems for flavonoid recovery has attracted considerable interest. AGREEprep™ is the first instrument specifically developed to assess the greenness of analytical sample preparation, applying ten criteria that together evaluate multiple facets of sample preparation sustainability [26,27].

Chromatographic profiling—using techniques such as HPLC-PDA, UHPLC-MS/MS and preparative chromatography—remains the primary approach for qualitative and quantitative characterization of flavonoid composition. Integrating HSP-guided solvent selection with rigorous chromatographic methods yields a workflow that concurrently enhances extraction efficiency and analytical resolution, thereby enabling precise comparison of flavonoid distribution across plant organs and evaluation of solvent selectivity for specific flavonoid subclasses [6,7,8].

This study integrates HSP theory with experimental green-solvent extraction and chromatographic profiling to: (1) predict and select environmentally preferable solvents for efficient flavonoid extraction from *O. indicum*; (2) assess the extraction performance of HSP-guided solvents relative to conventional solvents in terms of yield, selectivity and extract composition; (3) generate a detailed chromatographic fingerprint of flavonoid distribution across principal plant organs; and (4) assess the greenness of the sample-preparation procedure using the AGREEprep™ metric. By combining predictive solvent screening with empirical validation and comprehensive chromatographic characterization, the research aims to establish a reproducible, environmentally sustainable extraction protocol and to provide a reference dataset valuable for natural-product research, quality control and potential industrial utilization of *O. indicum*.

## 2. Results

### 2.1. Identification of Flavonoids in Oroxylum indicum Seed Extracts

**Figure 1:**
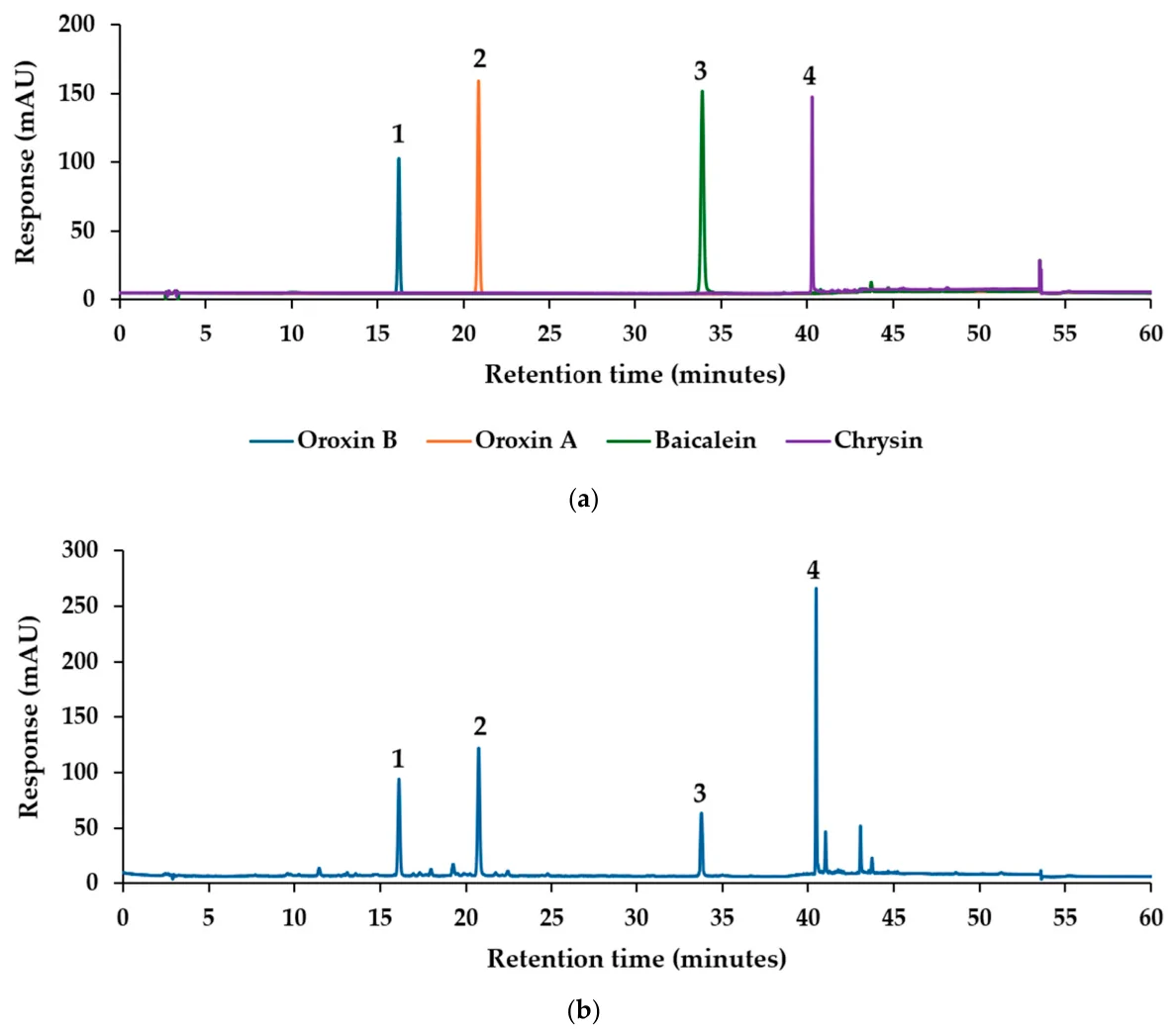

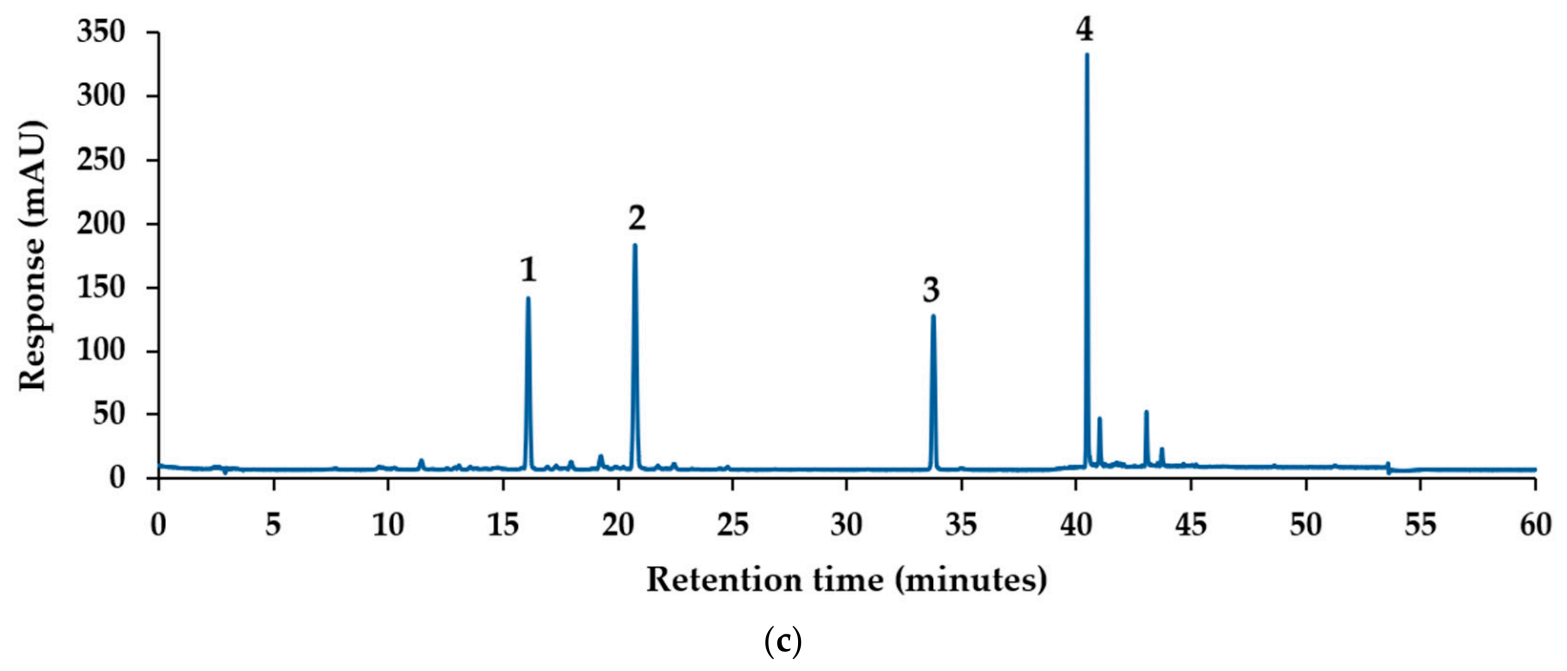
Chromatograms recorded at 276 nm of (**a**) the overlaid reference standards—oroxin B (1), oroxin A (2), baicalein (3), and chrysin (4); (**b**) the *O. indicum* seed extract; and (**c**) the *O. indicum* seed extract spiked with the corresponding reference standards.

High-performance liquid chromatography coupled with photodiode array detection (HPLC–PDA) was employed to identify flavonoids in *O. indicum* samples. As shown in Figure 1, the retention times of oroxin B, oroxin A, baicalein, and chrysin in both the seed extract and standard-addition samples were approximately 16.2, 20.8, 32.9, and 40.5 min, respectively. This elution order was consistent with the relative topological polar surface areas (TPSAs) reported in Table S1 and with previously published literature [6,7,8].

**Figure 2:**
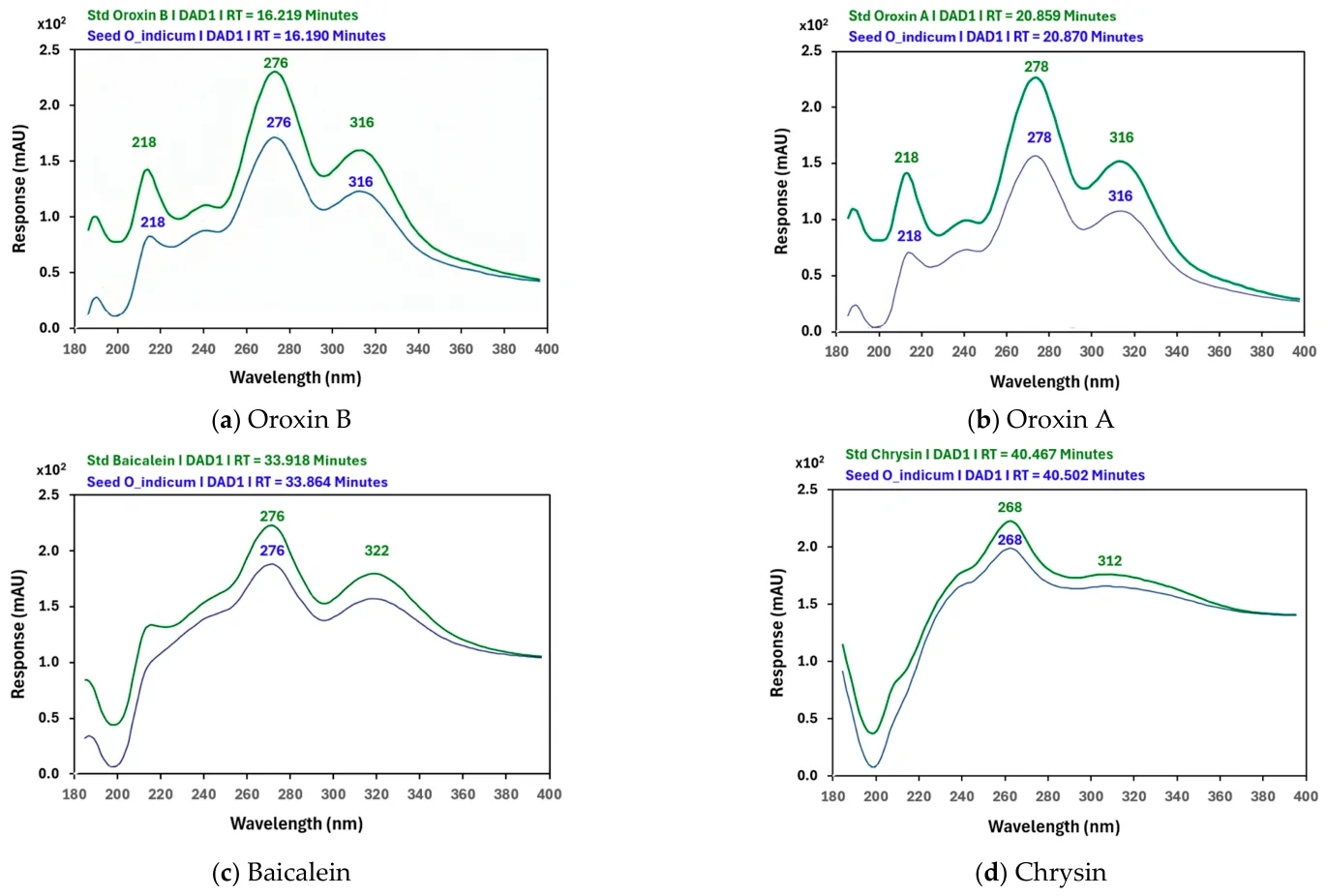
Ultraviolet spectra of the HPLC peaks of the *O. indicum* extract (blue) corresponding to the reference standards (green): (**a**) oroxin B, (**b**) oroxin A, (**c**) baicalein, and (**d**) chrysin.

The UV absorption profiles and maximum absorption wavelengths of the peaks eluting at 16.2, 20.8, 32.9, and 40.5 min were 276, 278, 276, and 268 nm, respectively. These chromatographic and spectral characteristics supported the identification of the corresponding compounds as oroxin B, oroxin A, baicalein, and chrysin, respectively (Figure 2). Because most of the identified compounds exhibited maximum UV absorption close to 276 nm, this wavelength was selected for the detection of all analytes in the present study.

### 2.2. Validation of HPLC Analytical Method for Determination of Flavonoids in Oroxylum indicum Extracts

The analytical method for the simultaneous determination of four flavonoids in *O. indicum* extracts was developed as described in Section 4.4. To ensure its suitability and reliability, the method was evaluated through system suitability testing and method validation in accordance with the International Council for Harmonisation (ICH) guidelines [28] and the AOAC Guidelines (2016) [29].

The analysis was conducted using a 60% ethanol extract of *O. indicum* seeds. Six replicate injections were performed under the specified high-performance liquid chromatography (HPLC) conditions, with an injection volume of 10 µL. As presented in Table 1, the system suitability results for all analytes met the established acceptance criteria, with relative standard deviations (%RSDs) for retention time and peak area below 2%. The number of theoretical plates exceeded 2500 for every injection, while the tailing factor remained below 1.08. Theoretical plate number is a direct indicator of chromatographic column efficiency; a higher value reflects improved separation performance and produces sharper, more clearly defined analyte peaks. In addition, the chromatographic resolution was greater than 2.0.

The HPLC–PDA method was validated in accordance with AOAC guidelines by assessing its linearity, limits of detection (LOD) and quantification (LOQ), precision, and accuracy. Linearity was evaluated using five-point calibration curves, with concentration levels selected according to the expected analyte concentrations in the *O. indicum* extracts, as determined from preliminary investigations.

As presented in Table 2, the calibration curves demonstrated excellent linearity for all reference standards over the concentration range of 1–50 µg/mL, with coefficients of determination (R^2^) ranging from 0.9997 to 1.0000. The LOD values for the four investigated marker compounds ranged from 0.13 to 0.31 µg/mL, whereas the corresponding LOQ values ranged from 0.39 to 0.94 µg/mL (Table 3).

The recovery rates for Compounds 1–4 ranged from 96.73% to 103.43%, as presented in Table 3. These values were within the acceptable range of 95–105% specified by the AOAC Guideline (2016) [29], indicating satisfactory method accuracy. Precision was assessed based on the relative standard deviation (RSD). The RSD values obtained for repeatability, as well as intra-day and inter-day precision, were all below 2.00% for the evaluated flavonoids. These results demonstrate that the method exhibited satisfactory precision and met the established validation criteria (Table 3). Overall, the validation findings confirm that the developed HPLC assay is accurate, precise, and suitable for the quantitative determination of the selected flavonoids in *O. indicum* extracts for quality-control purposes.

### 2.3. Solvent Selection for the Extraction of Flavonoids from Oroxylum indicum

The extraction of flavonoids from *O. indicum* requires the judicious selection of solvents to maximize extraction efficiency while minimizing toxicity, particularly when the extracts are intended for oral administration. Environmental impact and sustainability must also be considered. Solvent selection is influenced by several factors, including the polarity of the target flavonoids, the effectiveness of the extraction technique, and the physicochemical properties of the candidate solvents.

The Hansen solubility parameter (HSP) provides a comprehensive framework for evaluating solute–solvent compatibility based on three principal intermolecular interactions: dispersion forces (δD), dipolar interactions (δP), and hydrogen bonding (δH). Together, these parameters characterize the molecular interactions governing the dissolution of flavonoids in a given solvent. The calculated δD, δP, and δH values for oroxin B, oroxin A, baicalein, and chrysin are presented in Table 4.

Various ethanol–water ratios were evaluated for the extraction process using ultrasonic-assisted extraction. The Hansen solubility parameter distance (Ra) between the solute and solvent is an important consideration in selecting an appropriate solvent for extracting flavonoids from *O. indicum*. This parameter reflects the compatibility between solutes and solvents based on their dispersion, polar, and hydrogen-bonding interactions. Lower Ra values generally indicate greater miscibility and stronger molecular interactions between the solute and solvent [30].

As shown in Table 4, the Ra values of oroxin B, oroxin A, baicalein, and chrysin in water were 22.98, 14.69, 19.36, and 24.64, respectively. In ethanol, the corresponding values were 40.94, 25.16, 14.95, and 11.16. For oroxin B, the lowest Ra value was observed in water (0% ethanol; Ra = 22.98), indicating that the addition of ethanol did not improve its predicted solubility. In contrast, the ethanol–water mixtures generally produced lower Ra values than either pure solvent for the other compounds. The minimum Ra values were obtained at 10% ethanol for oroxin A (Ra = 14.34), 60% ethanol for baicalein (Ra = 12.07), and 90% ethanol for chrysin (Ra = 11.01).

These results suggest that the flavonoid glycosides, particularly oroxin A and oroxin B, may be more soluble in solvents containing relatively low or no ethanol, whereas the aglycones, baicalein and chrysin, may show greater solubility in solvents with higher ethanol concentrations. However, the experimental extraction results presented in Table 5 showed that most compounds were extracted more efficiently using solvent mixtures containing 60% or 80% ethanol. The highest flavonoid contents were obtained for oroxin B with 80% ethanol (65.18 ± 1.09 mg/g dry weight), oroxin A with 80% ethanol (128.38 ± 1.88 mg/g dry weight), baicalein with 60% ethanol (53.47 ± 1.07 mg/g dry weight), and chrysin with 60% ethanol (28.11 ± 0.68 mg/g dry weight).

The extraction results were consistent with the lower Ra values observed for the aglycones baicalein and chrysin but did not correspond to the predicted solubility trends for the flavonoid glycosides oroxin A and oroxin B. In particular, oroxin B exhibited its lowest Ra value in water, whereas its highest content was obtained with 80% ethanol. This discrepancy indicates that Ra values alone may not adequately predict extraction efficiency. Other factors, including ultrasonic cavitation, disruption of the plant matrix, solvent penetration, compound–matrix interactions, and compound stability, may also influence the extraction process [31,32]. Overall, ultrasound-assisted extraction of flavonoids from *O. indicum* seeds appears to be most effective with 60–80% ethanol, despite the lower predicted solubility of some glycosides in these solvent systems.

### 2.4. Chromatographic Profiling of Flavonoids in Different Plant Organs of Oroxylum indicum

**Figure 3:**
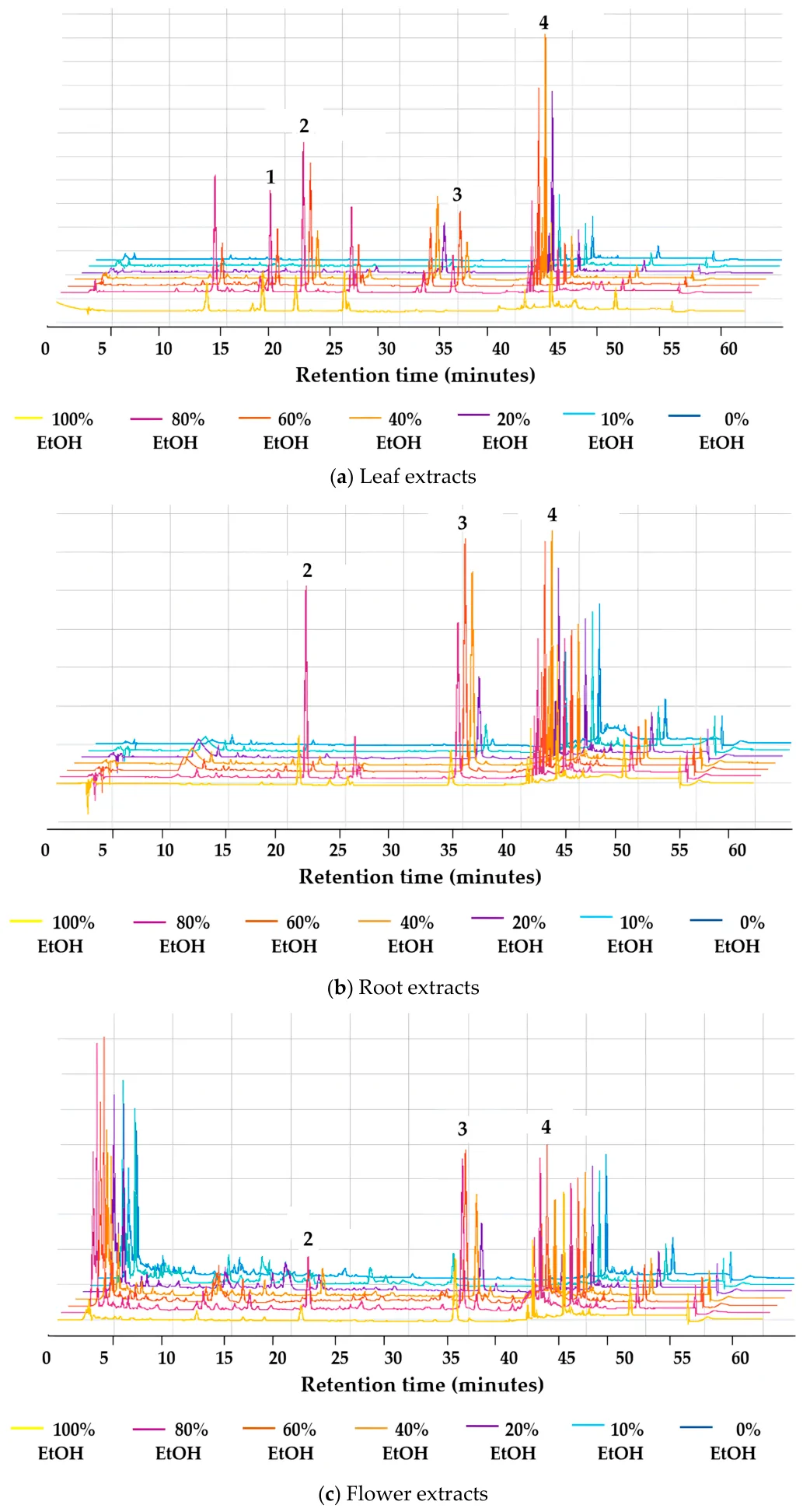

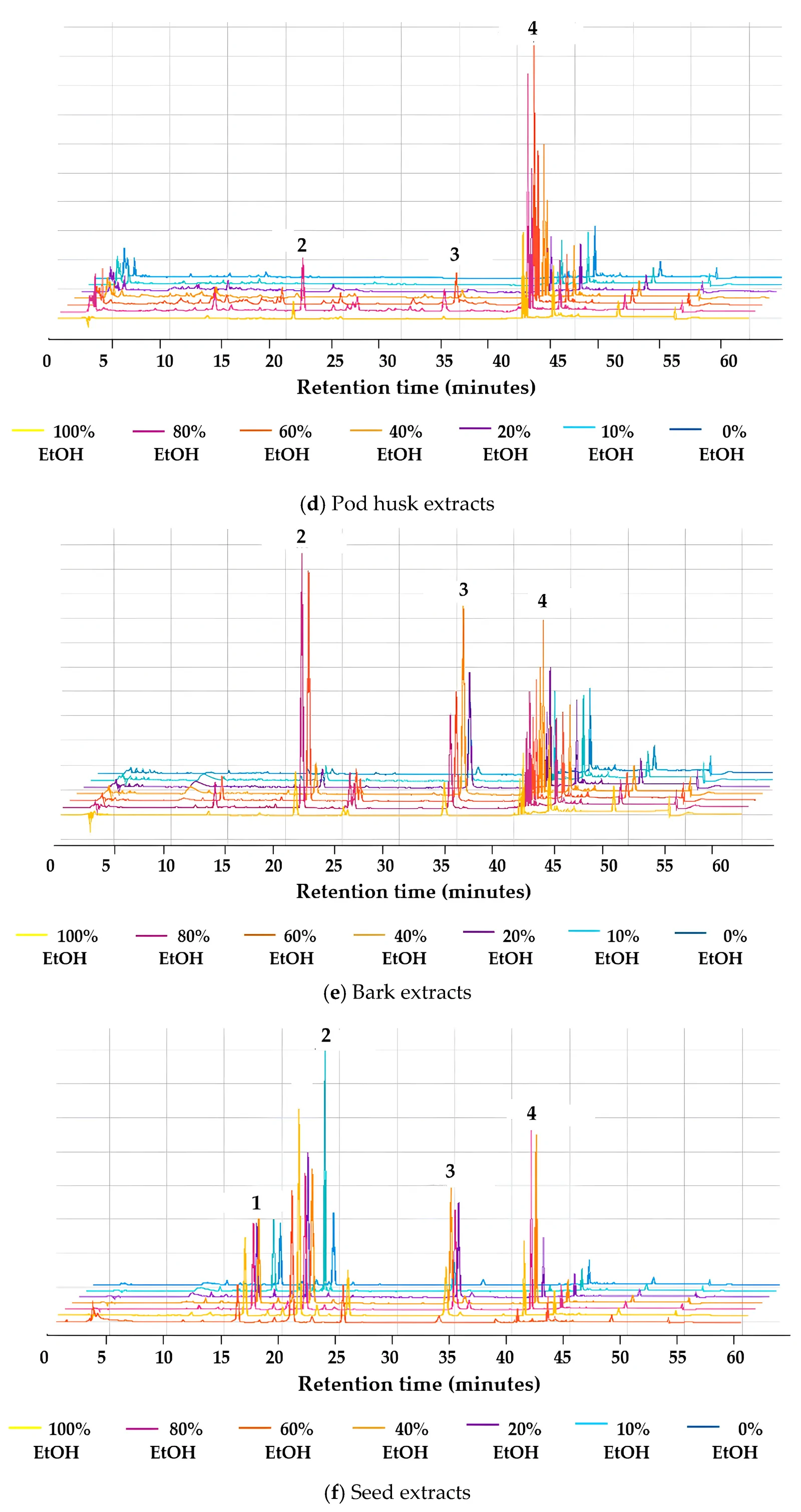
Chromatographic profiles of flavonoids in different solvent extracts of *O. indicum*: (**a**) leaves, (**b**) roots, (**c**) flowers (**d**) pod husks, (**e**) barks, and (**f**) seeds. Peaks 1–4 correspond to oroxin B, oroxin A, baicalein, and chrysin, respectively.

Six plant organs of *O. indicum*—namely, the leaves, roots, flowers, pod husks, bark, and seeds (Figure S2)—were extracted using seven ethanol–water solvent systems containing 0%, 10%, 20%, 40%, 60%, 80%, and 100% ethanol. The extracts were analyzed by HPLC–PDA at a UV detection wavelength of 276 nm. The resulting chromatographic profiles were processed using Agilent 1260 Infinity II software and are presented in Figure 3. Four characteristic flavonoid peaks—oroxin B, oroxin A, baicalein, and chrysin—were identified at retention times of 16.2, 20.8, 32.9, and 40.5 min, respectively.

### 2.5. Major Flavonoid Contents in Different Plant Organs of Oroxylum indicum

**Figure 4:**
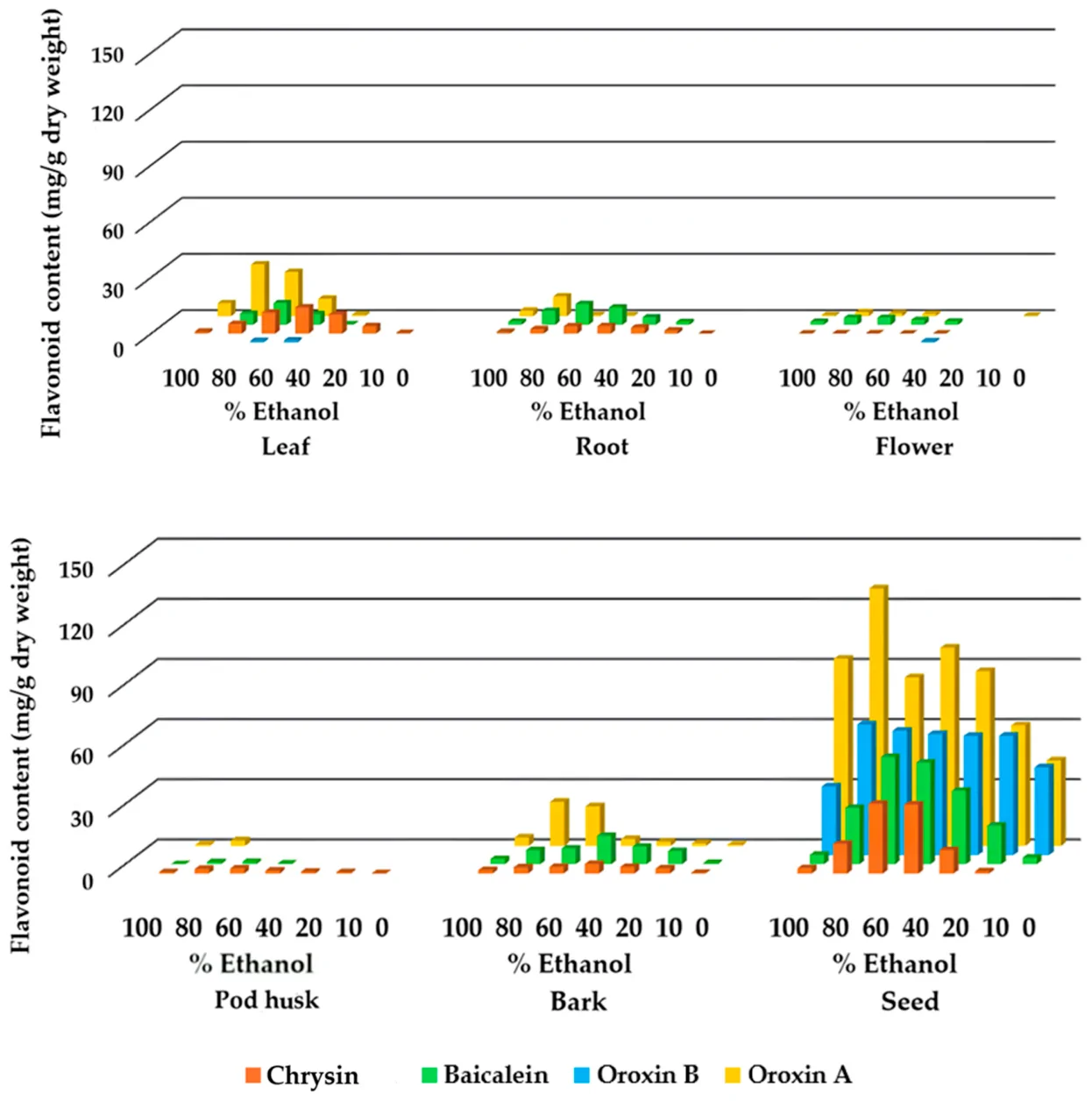
Oroxin B, oroxin A, baicalein, and chrysin contents (mg/g dry weight) in different solvent extracts and different organs of *O. indicum* (leaves, roots, flowers, pod husks, bark, and seeds.

The contents of oroxin B, oroxin A, baicalein, and oroxylin A differed considerably among the leaves, roots, flowers, pod husks, bark, and seeds (Figure 4). The results also demonstrated a strong effect of ethanol concentration on flavonoid recovery. Overall, the seeds contained substantially higher levels of all four flavonoids than the other plant organs, indicating that seeds are the richest source of the investigated flavonoids.

Oroxin B was detected predominantly in seed extracts, whereas only trace amounts were detected in the leaf and flower extracts. In the seed extracts, the oroxin B content increased from 34.21 mg/g dry weight in 100% ethanol to a maximum of 65.18 mg/g dry weight in 80% ethanol. Thereafter, the content decreased slightly with decreasing ethanol concentration, reaching 62.04 mg/g dry weight at 60% ethanol, 60.37 mg/g dry weight at 40% ethanol, 59.62 mg/g dry weight at 20% ethanol, 59.46 mg/g dry weight at 10% ethanol, and 43.75 mg/g dry weight in the aqueous extract. In the leaf extracts, oroxin B was detected at concentrations of 0.80 and 1.07 mg/g dry weight in the 60% and 40% ethanol extracts, respectively. In the flower extracts, it was detected at 0.64 mg/g dry weight in the 20% ethanol extract but was not detected in the root, pod-husk, or bark extracts. These findings indicate that oroxin B preferentially accumulates in seeds and that 80% ethanol is the most effective solvent concentration for extracting this compound from this organ.

Oroxin A was detected in all six plant organs and was generally more abundant than the other flavonoids, particularly in seeds. The highest oroxin A content was observed in the seed extract prepared with 80% ethanol, reaching 128.38 mg/g dry weight. The corresponding contents were 93.32, 104.02, 98.79, 87.12, 60.11, and 42.56 mg/g dry weight in extracts prepared with 100%, 60%, 40%, 20%, and 10% ethanol and water, respectively. Among the non-seed organs, the highest oroxin A contents were observed in the leaves and bark. Leaf extracts contained 27.60 and 23.59 mg/g dry weight when prepared with 80% and 60% ethanol, respectively, whereas bark extracts contained 21.95 and 19.71 mg/g dry weight under the same conditions. The root extract reached a maximum oroxin A content of 10.51 mg/g dry weight with 80% ethanol. Lower concentrations were detected in the flowers and pod husks, with maximum values of 2.08 and 2.71 mg/g dry weight, respectively, both obtained using 80% ethanol.

Baicalein exhibited a distribution pattern distinct from those of the glycosylated flavonoids. The highest baicalein content was obtained from the seed extract prepared with 60% ethanol, reaching 53.47 mg/g dry weight. A comparable content was observed in the 40% ethanol extract (50.71 mg/g dry weight), whereas lower contents were obtained with 20% ethanol (36.61 mg/g dry weight), 10% ethanol (19.28 mg/g dry weight), and water (3.32 mg/g dry weight). The 80% and 100% ethanol extracts yielded 28.09 and 4.73 mg/g dry weight, respectively. Among the non-seed organs, bark was the richest source of baicalein. Its highest content, 14.14 mg/g dry weight, was obtained with 40% ethanol, followed by 8.78, 7.80, 7.01, 6.55, 2.62, and 0.61 mg/g dry weight in extracts prepared with 20%, 60%, 80%, 10%, and 100% ethanol and water, respectively. In the root extracts, the maximum baicalein content was 11.10 mg/g dry weight with 60% ethanol, whereas the leaf extracts yielded a maximum content of 11.64 mg/g dry weight under the same conditions. Flowers and pod husks contain considerably lower amounts, with maximum contents of 3.88 and 1.19 mg/g dry weight, respectively.

Chrysin was detected in most plant-organ extracts and occurred at its highest concentration in the seeds. The maximum content in the seed extracts was 28.11 mg/g dry weight with 60% ethanol, closely followed by 27.80 mg/g dry weight with 40% ethanol. The content decreased to 11.94, 9.45, 2.16, and 0.88 mg/g dry weight in extracts prepared with 80%, 20%, 100%, and 10% ethanol, respectively. Chrysin was not detected in the aqueous seed extract. In the leaf extracts, the highest chrysin content was 11.34 mg/g dry weight with 40% ethanol, followed by 9.03 and 8.35 mg/g dry weight with 60% and 20% ethanol, respectively. The root and bark extracts showed maximum contents of 3.22 and 3.85 mg/g dry weight, respectively, both obtained with 40% ethanol. In the pod-husk extracts, the highest content was 2.01 mg/g dry weight with 60% ethanol, whereas the flowers contained only trace amounts, with a maximum of 0.14 mg/g dry weight obtained with both 60% and 80% ethanol.

Overall, the optimum ethanol concentration depended on both the target flavonoid and the plant organ. The highest concentrations of all four compounds were obtained from the seed extracts, although the optimal solvent concentration varied among the compounds:

Oroxin B: 65.18 ± 1.09 mg/g dry weight in seeds extracted with 80% ethanol.

Oroxin A: 128.38 ± 1.88 mg/g dry weight in seeds extracted with 80% ethanol.

Baicalein: 53.47 ± 1.07 mg/g dry weight in seeds extracted with 60% ethanol.

Chrysin: 28.11 ± 0.68 mg/g dry weight in seeds extracted with 60% ethanol.

The flavonoid concentrations in the other plant organs were generally much lower than those in the seeds. The glycosylated flavonoids, oroxin B and oroxin A, were extracted more effectively with 80% ethanol, whereas the aglycones, baicalein and chrysin, showed higher recoveries at moderate ethanol concentrations of 40–60%. These differences may primarily be attributed to solvent polarity, as well as to solvent penetration, disruption of the plant matrix, and ultrasound-assisted mass transfer. Thus, the optimum ethanol concentration varied according to both the target flavonoid and the plant organ.

### 2.6. Assessing the Environmental Sustainability of the Extraction Methods

**Figure 5:**
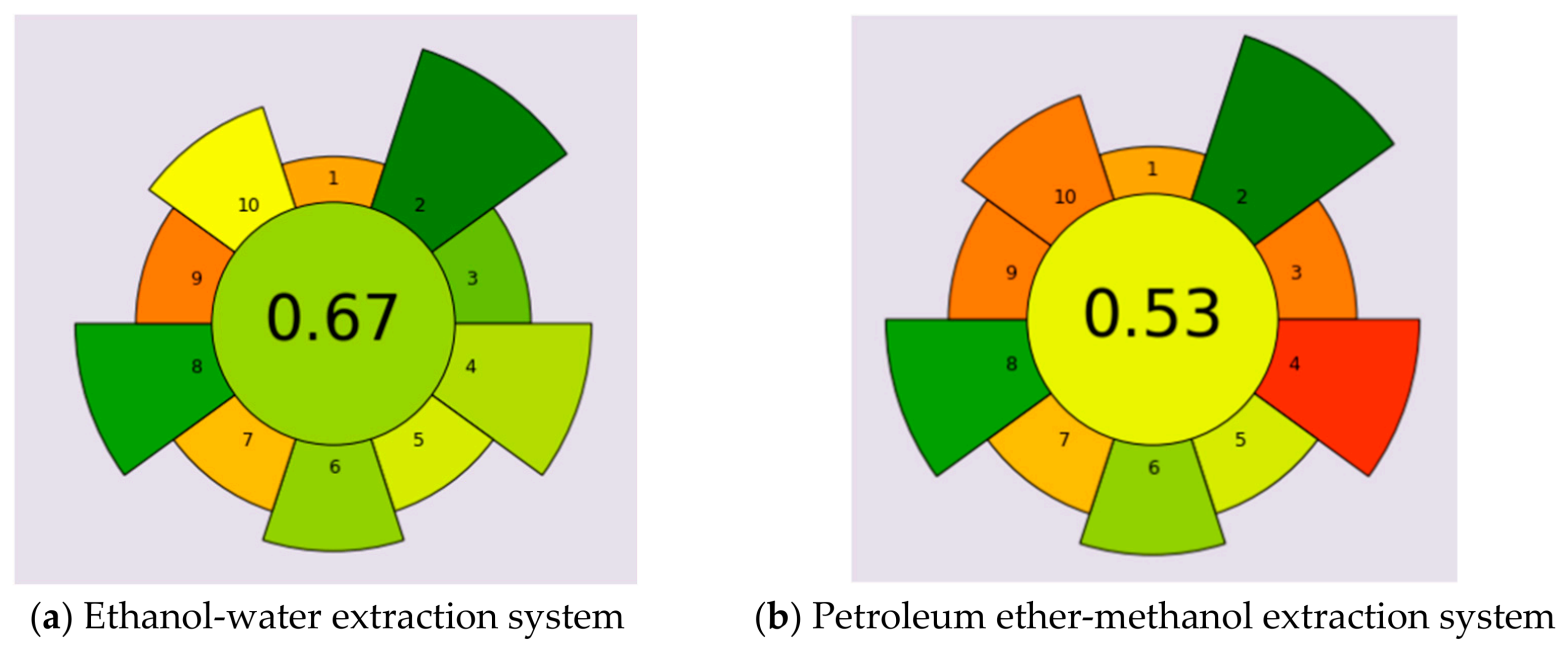
Comparison of the greenness assessment of flavonoid-extraction methods using (**a**) an ethanol–water solvent system and (**b**) a petroleum ether–methanol solvent system. The AGREEprep criteria comprise: (1) sample preparation placement; (2) hazardous materials/solvents; (3) sustainability, renewability, and reusability; (4) waste minimization; (5) sample size (size economy); (6) sample throughput; (7) integration and automation; (8) energy consumption; (9) post-sample preparation configuration; and (10) operator safety.

Petroleum ether and methanol are effective solvents for extracting relatively high amounts of flavonoids from *O. indicum*. However, both are volatile organic solvents that may pose risks to human health and the environment [33]. Therefore, these solvents were included in the assessment to enable a comparative evaluation of the environmental sustainability of different extraction methods using a petroleum ether–methanol system and an ethanol–water system, the latter being considered a greener solvent system. The greenness of the sample-preparation strategies used for flavonoid analysis was evaluated using the AGREEprep metric, as described in Section 4.6. AGREEprep is an analytical tool designed to assess the sustainability of sample-preparation procedures based on ten criteria and a penalty-point system. The resulting score ranges from 0 to 1, where 0 represents the least sustainable procedure and 1 indicates the highest level of greenness. Accordingly, higher AGREEprep scores reflect more environmentally sustainable procedures [26,27]. Figure 5 presents the AGREEprep assessment of the most promising flavonoid-extraction methods identified in Section 4.3 and Section 4.4. The ethanol–water extraction systems achieved a mean AGREEprep score of 0.67, whereas the petroleum ether–methanol systems obtained a lower mean score of 0.53. These findings demonstrate that the ethanol–water systems have a more favorable environmental profile than the petroleum ether–methanol systems. Thus, solvent selection plays an important role in determining the environmental sustainability of flavonoid-extraction procedures.

## 3. Discussion

The present study applied Hansen solubility parameters (HSPs) to guide the selection of ethanol–water solvent systems for the ultrasound-assisted extraction of four major flavonoids from *O. indicum*, which had been identified in a previous report [34,35]. The target compounds comprised two glycosides, oroxin B and oroxin A, and two aglycones, baicalein and chrysin. Their structural differences substantially influenced their physicochemical properties and extraction behavior. Oroxin B, a disaccharide glycoside of baicalein, was the most hydrophilic compound, followed by the monosaccharide glycoside oroxin A. In contrast, baicalein and chrysin, a 6-dehydroxylated derivative of baicalein, were less polar. The predicted aqueous solubility therefore decreased in the order oroxin B > oroxin A > baicalein > chrysin. This trend was consistent with the differences in calculated logP and total polar surface area and provided an initial basis for understanding solvent–solute interactions.

For HPLC-PDA analysis, total run time of this study was 60 min longer than previous research (30 min) [35], because this study aimed to characterize the chromatographic profiles of six tissues of *O. indicum*: seeds, leaves, roots, bark, pod husks, and flowers. Compared with the fruit analyzed in a previous study, these tissues exhibited more complex chromatographic profiles and a greater number of interfering peaks. Moreover, the present study employed a broader range of solvent compositions, which may have affected the types of compounds detected. Accordingly, the total analysis time was extended to 60 min to facilitate the separation of the interfering peaks from the four target compounds. In addition, the detection wavelength was selected to closely approximate the maximum absorption wavelengths of the four target compounds. Accordingly, a wavelength of 276 nm was used in the present study, resulting in low limits of detection (LODs; 0.13–0.31 µg/mL) and limits of quantification (LOQs; 0.31–0.93 µg/mL), whereas the previous study employed a wavelength of 285 nm.

HSP analysis provided useful information regarding the compatibility between each flavonoid and the tested solvents. The Hansen solubility parameter distance (Ra) describes the difference between solute and solvent in terms of dispersion, polar, and hydrogen-bonding interactions. In general, a lower Ra value indicates greater predicted compatibility and stronger intermolecular interactions [13]. The calculated values showed that oroxin B had its lowest Ra value in water, whereas the lowest values for oroxin A, baicalein, and chrysin occurred in 10%, 60%, and 80% ethanol, respectively. These results suggested that the glycosides should be favored by highly aqueous systems, while the aglycones should be more compatible with solvents containing higher proportions of ethanol.

However, the experimental extraction results demonstrated that Ra values alone could not fully predict extraction efficiency. The highest recovery of oroxin B was obtained using 80% ethanol, although its lowest Ra value was observed in water. Similarly, oroxin A showed its highest content in 80% ethanol despite a lower predicted Ra value in 10% ethanol. These findings indicate that extraction is governed by multiple processes in addition to equilibrium solubility. During ultrasound-assisted extraction, acoustic cavitation generates localized high-energy conditions that promote cell-wall disruption, tissue swelling, solvent penetration, and enhanced mass transfer [24]. The ethanol–water ratio may therefore affect not only flavonoid solubility but also the accessibility of intracellular compounds and their interactions with other matrix components. Ethanol may additionally improve membrane permeability and reduce the strength of compound–matrix associations, thereby increasing the release of glycosylated flavonoids from the plant tissue [25].

The extraction behavior of the aglycones was more consistent with the HSP predictions. Baicalein reached its highest content in 60% ethanol, while chrysin showed its highest content at the same concentration. These intermediate ethanol concentrations provided a favorable balance between the dissolution capacity of ethanol and the hydration and swelling effects of water. Although absolute ethanol may improve the solubility of less-polar compounds, its limited water content may reduce tissue swelling and solvent diffusion into the plant matrix. Conversely, water alone may not provide sufficient solvent strength for efficient dissolution of the aglycones. Thus, the superior performance of 60% ethanol likely resulted from the combined effects of solvent polarity, matrix hydration, cavitation, and mass transfer [24,25].

The four flavonoids showed distinct organ-specific distribution patterns among the seeds, leaves, roots, flowers, pod husks, and bark of *O. indicum* (Figure 3). Seeds contained substantially higher concentrations of all four compounds than the other plant organs, supporting their importance as the principal reservoir of the investigated flavonoids. This result is consistent with previous phytochemical reports identifying the seeds of *O. indicum* as a particularly rich source of flavonoids and flavonoid glycosides, although the relative abundance of individual compounds may vary according to the plant material, geographic origin, developmental stage, and extraction conditions [36,37].

Oroxin B exhibited the most restricted distribution. It was detected predominantly in the seeds, where its concentration reached 6.52 ± 0.11 mg/g dried weight with 80% etha-nol, but was present only at trace levels in the leaves and flowers and was not detected in the roots, pod husks, or bark. This pattern suggests preferential accumulation of oroxin B in seed tissues. In contrast, oroxin A was detected in all six organs and was the most abundant compound overall, particularly in the seeds. Its highest concentration was 12.84 ± 0.19 mg/g dried weight in the seed extract obtained with 80% ethanol. Among the non-seed organs, leaves and bark contained the highest levels, whereas flowers and pod husks contained only small amounts. The broader distribution of oroxin A compared with oroxin B may indicate differences in biosynthesis, transport, or tissue-specific storage.

Baicalein and chrysin showed distribution patterns that differed from those of the glycosylated flavonoids. Baicalein was most abundant in seeds, reaching 5.35 ± 0.11 mg/g dried weight with 60% ethanol, while bark was the richest non-seed source, followed by roots and leaves. Chrysin was also concentrated mainly in the seeds, with a maximum content of 2.81 ± 0.07 mg/g dried weight at 60% ethanol. Among the non-seed organs, leaves contained the highest level of chrysin, followed by bark, roots, and pod husks; only trace amounts were detected in flowers. The relatively high levels of baicalein and chrysin in bark and leaves may reflect the protective role of these tissues, where flavonoid agly-cones can contribute to defense against ultraviolet radiation, oxidative stress, and herbivory. Conversely, the low concentrations in flowers and pod husks suggest that these organs are less important sources of the four target compounds under the conditions examined.

The extraction results further demonstrated that solvent composition strongly influenced flavonoid recovery. The optimum ethanol concentration was compound- and organ-dependent: 80% ethanol gave the highest recoveries of oroxin B and oroxin A from seeds, whereas 60% ethanol was optimal for baicalein and chrysin. This variation may be related to differences in polarity, glycosylation, and interactions between the flavonoids and the plant matrix. Overall, the results agree with the previously described phytochemical richness of *O. indicum* seeds while also demonstrating that leaves and bark may serve as useful alternative sources of selected flavonoids, particularly oroxin A, baicalein, and chrysin [38,39,40].

Finally, the sustainability assessment confirmed the environmental advantage of the ethanol–water system. Although petroleum ether and methanol can efficiently extract flavonoids, their toxicity, volatility, and environmental hazards limit their suitability for sustainable sample preparation. The ethanol–water systems achieved a mean AGREEprep score of 0.67, compared with 0.53 for the petroleum ether–methanol systems. The higher score indicates a more favorable greenness profile [26,27].

Overall, the findings demonstrate that ethanol–water mixtures, combined with ultrasound-assisted extraction, provide an effective and comparatively sustainable approach for recovering flavonoids from *O. indicum*. The integration of HSP-based solvent assessment, chromatographic validation, organ-specific profiling, and greenness evaluation offers a practical framework for developing efficient and environmentally responsible extraction methods.

Future research should further clarify the relationship between Hansen solubility parameter predictions and the actual extraction behavior of flavonoids from *O. indicum*. Although the present study demonstrated that HSP-based R_a_ values provide useful preliminary information, their predictive capacity was not sufficient to explain the extraction yields of all compounds, particularly the glycosides. Subsequent studies should therefore integrate HSP calculations with experimental measurements of solubility, partition coefficients, desorption behavior, and solvent–matrix interactions. The use of experimentally determined HSPs for the major flavonoids may also improve the accuracy of solvent-selection models.

The biological relevance of the extracts should be evaluated alongside their chemical composition. Extracts prepared under different solvent conditions could be compared for antioxidant, anti-inflammatory, antimicrobial, cytotoxic, and other pharmacological activities. Correlation analysis between individual flavonoid concentrations and biological responses would help determine whether activity is primarily associated with specific compounds or with synergistic interactions within the extracts. However, such studies should include appropriate toxicity, selectivity, and mechanism-of-action assessments before any therapeutic claims are made.

The sustainability assessment should also be expanded beyond the AGREEprep score. Future investigations should conduct a comprehensive life-cycle assessment involving solvent production, water consumption, electricity use, ultrasound energy, waste generation, solvent recycling, and greenhouse-gas emissions. Green analytical metrics such as analytical eco-scale, GAPI, and complementary process-level indicators could be used to provide a more complete sustainability profile [41,42]. The feasibility of replacing ethanol with bio-based solvents or deep eutectic solvents should also be examined, provided that their toxicity, biodegradability, viscosity, recovery, and compatibility with downstream applications are carefully evaluated.

## 4. Materials and Methods

### 4.1. Materials

Plant material was collected from the Botanical Garden, Faculty of Pharmacy, Srinakharinwirot University, Nakhon Nayok, Thailand. Ethanol, chromatography-grade acetonitrile and methanol were purchased from Merck (Darmstadt, Germany), and formic acid was obtained from Sigma-Aldrich Co. (St. Louis, MO, USA). All reference standards were supplied by ChemFaces (Wuhan, China).

### 4.2. Calculation of Hansen Solubility Parameters

The Hansen solubility parameters (HSPs) for flavonoids were estimated using the group contribution approach of Stefanis et al. [13], applied to the compounds’ molecular structures. The dispersion force (δD), dipolar intermolecular force (δP), and hydrogen bonding energy (δH) values were calculated using Equations (1)–(3), respectively.

(1) $$\delta D=\left(\sum_{i}N_{i}C_{i}+W\sum_{j}M_{j}D_{j}+17.3231\right){\mathrm{MPa}}^{1/2}$$

(2) $$\delta P=\left(\sum_{i}N_{i}C_{i}+W\sum_{j}M_{j}D_{j}+7.3548\right){\mathrm{MPa}}^{1/2}$$

(3) $$\delta H=\left(\sum_{i}N_{i}C_{i}+W\sum_{j}M_{j}D_{j}W+7.9793\right){\mathrm{MPa}}^{1/2}$$

In these equations, Ci denotes the contribution of a first-order group of type i occurring Ni times in the molecule, and Dj denotes the contribution of a second-order group of type j occurring Mj times. The binary constant W equals 0 for compounds lacking second-order groups and 1 for those that contain them. The δD, δP, and δH values of the mixed-solvent system were calculated using a simple volume-fraction (or volume-percentage)-weighted average of the corresponding parameters of the individual pure solvents. Solvents whose HSP components closely match those of the flavonoids were chosen for extraction. Solvent–solute compatibility was quantified by the interaction radius (Ra), computed using Equation (4).

(4) $$\mathrm{Ra}=\sqrt{4{\left(\delta_{\mathrm{Di}}-\delta_{\mathrm{Dj}}\right)}^{2}+{\left(\delta_{\mathrm{Ppi}}-\delta_{\mathrm{Pj}}\right)}^{2}+{\left(\delta_{\mathrm{Hi}}-\delta_{\mathrm{Hj}}\right)}^{2}}$$

where i refers to the HSPs of the solute, and j refers to the HSPs of the solvent.

### 4.3. Preparation of Herbal Extract

The six herbal components were dried in a hot-air oven at ambient pressure for 24 h, then finely pulverized using an herbal grinder and passed through a 40-mesh sieve. Extractions were performed with ethanol, water, and a water–ethanol mixture as solvents. For each extraction, 1 g of the powdered sample was mixed with the extraction solvent at a solid-to-solvent ratio of 1:25 (*w*/*v*). The mixture was sonicated for 20 min in an ultrasonic bath operating at 40 kHz and 300 W, with the temperature maintained at 40 °C. Following sonication, samples were centrifuged at 10,000 rpm for 15 min, and the resulting clear supernatants were collected and stored at 2–8 °C until further analysis.

### 4.4. HPLC-PDA Analysis

High-performance liquid chromatography (HPLC) analyses were carried out using an Agilent 1260 Infinity II system (Santa Clara, CA, USA) equipped with a quaternary pump, autosampler, column thermostat, and photodiode array detector. Chromatographic separation was performed on an ACE C18-AR column (4.6 × 250 mm, 5 µm; Avantor, Aberdeen, UK) with a Phenomenex C18 guard column (4 × 3 mm, 5 µm; Torrance, CA, USA). The mobile phase comprised 0.1% formic acid in ultrapure water (solvent A) and 0.1% formic acid in acetonitrile (solvent B). A gradient elution was applied: 15 → 40% B over 35 min, increased to 99% B within 5 min, held at 99% B for 10 min, returned to 15% B over 3 min, and re-equilibrated at 15% B for 7 min. The flow rate was 1.0 mL/min, column temperature 25 °C, and injection volume 10 µL. Detection was performed by PDA scanning from 200 to 600 nm, with quantitative monitoring at 276 nm. The developed analytical method was subjected to system suitability testing and validated in accordance with International Council for Harmonisation (ICH) requirements and the AOAC Guideline (2016) [29].

### 4.5. Method Validation

The HPLC-PDA method was validated in accordance with ICH [28] and AOAC [29] guidelines, evaluating linearity, limit of detection (LOD), limit of quantification (LOQ), accuracy, and precision.

#### 4.5.1. Specificity

The specificity of the HPLC–PDA method was assessed by comparing chromatograms of mixed reference standards with those of the sample extracts at 276 nm. Peaks corresponding to target compounds were confirmed as baseline-resolved from adjacent chromatographic peaks based on retention times. Specificity was additionally validated by performing triplicate analyses of both the standards and the samples.

#### 4.5.2. Linearity

Five-point calibration curves (1–50 µg/mL) were constructed for each reference standard, exhibiting correlation coefficients (r) > 0.995 in accordance with AOAC guidelines. All measurements were performed in triplicate.

#### 4.5.3. Limit of Detection (LOD) and Limit of Quantification (LOQ)

The limits of detection (LOD) and quantification (LOQ) were derived from the linear regression parameters using the following expressions:

LOD = 3.3 × (SD of the y-intercept)/(mean slope)

LOQ = 10 × (SD of the y-intercept)/(mean slope)

Thus, LOD and LOQ were calculated as 3.3- and 10-fold multiples, respectively, of the ratio between the standard deviation of the calibration curve intercept and the mean slope.

#### 4.5.4. Accuracy

Method recovery was evaluated by spiking samples at three concentration levels: 15, 30, and 45 µg/mL for oroxin B, baicalein, and chrysin, and 15, 25, and 35 µg/mL for oroxin A. Percent recovery was determined by comparing measured concentrations to the nominal spiked amounts. Accuracy was reported as the mean recovery (%) from triplicate injections at each low, medium, and high level.

#### 4.5.5. Precision

Method precision was assessed in terms of repeatability and intermediate precision. Repeatability was determined from three consecutive injections performed on the same day. Intermediate precision was evaluated by conducting triplicate injections at low, medium, and high concentration levels across three separate days. Precision was expressed as the percent relative standard deviation (%RSD).

### 4.6. Sustainability Assessment of the Extraction Methods

AGREEprep™ version 1.0 open-access software [26] was employed to evaluate the environmental performance of the proposed extraction methods. Developed by Wojnowski et al. as a practical instrument for assessing the ecological impact of sample preparation, the tool examines ten impact categories and transforms them into normalized sub-scores on a 0–1 scale [26]. These sub-scores are aggregated to produce an overall assessment score, where 1 represents the most environmentally favorable method and 0 the least sustainable. Results are presented graphically via a pictogram. The evaluation framework is grounded in ten principles of green sample preparation (GSP) (Pena-Pereira et al., 2022) [27].

### 4.7. ADMET Prediction Assessment

ADMET profiles of four flavonoids were assessed using the online SwissADME platform (http://www.swissadme.ch; accessed 15 January 2026) [43]. The Simplified Molecular Input Line Entry System (SMILES) representations of all ligands were retrieved from the PubChem database and used as input for the analyses.

### 4.8. Statistical Analysis

The data are presented as the mean ± standard deviation (SD) and were analyzed using OriginPro 9.0 software. One–way analysis of variance (ANOVA) followed by Fisher’s LSD test (*p* < 0.05) was used to determine differences between groups.

## 5. Conclusions

This study demonstrated that Hansen solubility parameters provide a useful preliminary framework for selecting ethanol–water systems for the ultrasound-assisted extraction of flavonoids from *O. indicum*. However, extraction efficiency was influenced not only by solute–solvent compatibility but also by cavitation, matrix hydration, solvent penetration, mass transfer, and compound–matrix interactions. Glycosides, particularly oroxin B and oroxin A, were extracted most effectively with 80% ethanol, whereas the aglycones baicalein and chrysin showed optimum recovery with 60% ethanol. Seeds were the richest source of all four target flavonoids, while substantial organ-specific differences were observed across leaves, roots, flowers, pod husks, and bark. Chromatographic profiling confirmed the selective distribution of these constituents. The ethanol–water systems also demonstrated superior environmental performance, achieving a higher AGREEprep score than petroleum ether–methanol. Overall, the integrated HSP-guided, chromatographic, organ-specific, and green approach offers an efficient and sustainable strategy for valorizing *O. indicum*.

## Figures and Tables

**Table 1:** System suitability results of the analytical method.

Analyte	% RSD of Retention Time	% RSD of Peak Area	Resolution	Number of Theoretical Plate	Tailing Factor
Acceptance criteria	≤2%	≤2%	>2	2500	≤1.2
Oroxin B	0.08	0.15	5.58	89,851	±1.01
Oroxin A	0.35	0.22	6.54	125,794	±1.00
Baicalein	0.05	1.41	30.12	182,510	±1.08
Chrysin	0.01	0.32	4.03	2,243,973	±1.03

**Table 2:** The retention time, linear range, regression equation, coefficient of determination (R^2^), limit of detection (LOD), and limit of quantification (LOQ) for the simultaneous analysis of four reference standards by HPLC (*n* = 3).

Retention Time (Minutes)	Analyte	Range (µg/mL)	Linear Equation	R^2^	LOD (µg/mL)	LOQ (µg/mL)
			Y = 27.589X + 3.329 Y = 27.572X + 7.049 Y = 27.529X + 6.512	1.0000	0.24	0.73
16.2	Oroxin B	1–50		0.9999		
				0.9999		
			Mean slope = 27.563	SD intercept = 2.0107		
			Y = 36.200X + 2.329 Y = 36.354X + 8.486 Y = 36.299X + 6.847	0.9998	0.29	0.88
20.8	Oroxin A	1–40		0.9998		
				0.9998		
			Mean slope = 36.284	SD intercept = 2.1887		
			Y = 56.077X Y = 55.051X Y = 54.600X	0.9997	0.31	0.94
32.9	Baicalein	1–50		0.9997		
				0.9997		
			Mean slope = 55.243	SD intercept = 5.1906		
			Y = 59.216X Y = 59.472X Y = 58.859X	0.9998	0.13	0.39
40.5	Chrysin	1–50		0.9997		
				0.9998		
			Mean slope = 59.182	SD intercept = 2.3346		

**Table 3:** Accuracy and precision for flavonoids in *O indicum* (*n* = 3).

Analyte	Spiked (µg/mL)	Day	Found (µg/mL)	SD	Intra-Day %RSD	%Recovery	Inter-Day %RSD
Oroxin B	15	1	15.04	0.10	0.69	100.27	0.55
		2	14.99	0.16	1.09	99.93	
		3	14.88	0.17	1.15	99.20	
	30	1	29.68	0.16	0.55	98.93	0.14
		2	29.76	0.09	0.30	99.20	
		3	29.73	0.06	0.19	99.10	
	45	1	43.53	0.10	0.24	96.73	0.92
		2	43.86	0.12	0.27	97.47	
		3	44.33	0.33	0.74	98.51	
Oroxin A	15	1	15.42	0.21	1.33	102.80	1.02
		2	15.24	0.13	0.84	101.60	
		3	15.11	0.12	0.76	100.73	
	25	1	25.54	0.12	0.47	102.16	1.50
		2	25.63	0.13	0.49	102.52	
		3	24.93	0.08	0.32	99.72	
	35	1	34.74	0.13	0.38	99.26	0.70
		2	35.22	0.12	0.34	100.63	
		3	35.06	0.09	0.25	100.17	
Baicalein	15	1	14.85	0.12	0.82	99.00	1.05
		2	14.61	0.11	0.72	97.40	
		3	14.90	0.14	0.97	99.33	
	30	1	31.03	0.30	0.97	103.43	1.77
		2	30.02	0.13	0.44	100.07	
		3	30.86	0.09	0.29	102.87	
	45	1	45.17	0.06	0.13	100.38	1.18
		2	46.17	0.15	0.33	102.60	
		3	46.03	0.13	0.29	102.29	
Chrysin	15	1	15.35	0.23	1.50	102.33	0.87
		2	15.22	0.15	0.99	101.47	
		3	15.09	0.36	2.39	100.60	
	30	1	30.62	0.44	1.44	102.07	1.33
		2	30.01	0.51	1.70	100.03	
		3	29.87	0.67	2.24	99.57	
	45	1	45.47	0.97	2.13	101.04	1.37
		2	45.99	1.05	2.28	102.20	
		3	44.76	0.88	1.97	99.47	

**Table 4:** Calculated Hansen solubility parameter distance (Ra) values between the flavonoids and aqueous–ethanol solvents.

Compounds/ Solvent	HSPs			Ra			
	δD	δP	δH	Oroxin B	Oroxin A	Baicalein	Chrysin
Oroxin B	17.57	1.30	59.47				
Oroxin A	18.61	2.72	43.16				
Baicalein	21.68	12.76	27.75				
Chrysin	21.15	11.66	20.79				
100% Ethanol	15.80	8.80	19.40	40.92	25.16	14.95	11.16
90% Ethanol	15.77	9.52	21.69	38.83	23.22	13.67	11.01
80% Ethanol	15.74	10.24	23.98	36.78	21.38	12.71	11.37
70% Ethanol	15.71	10.96	26.27	34.78	19.67	12.16	12.20
60% Ethanol	15.68	11.68	28.56	32.82	19.67	12.07	13.42
50% Ethanol	15.65	12.40	30.85	30.94	16.74	12.45	14.92
40% Ethanol	15.62	13.12	33.14	29.12	15.63	13.27	16.64
20% Ethanol	15.56	14.56	37.72	25.79	14.39	15.89	20.49
10% Ethanol	15.53	15.28	40.01	24.31	14.34	17.55	22.56
0% Ethanol	15.50	16.00	42.30	22.98	14.69	19.36	24.68

**Table 5:** Flavonoid contents (mg/g dry weight) of flavonoids from *O. indicum* seeds obtained using various aqueous–ethanol solvents (*n* = 3).

Solvents	Flavonoid Content (mg/g Dry Weight)			
	Oroxin B *	Oroxin A *	Baicalein *	Chrysin *
100% Ethanol	34.21 ± 0.42	93.32 ± 1.22	4.73 ± 0.23	2.16 ± 0.22
80% Ethanol	65.18 ± 1.09	128.38 ± 1.88	28.09 ± 0.78	11.94 ± 0.52
60% Ethanol	62.04 ± 0.81	104.02 ± 0.93	53.47 ± 1.07	28.11 ± 0.68
40% Ethanol	60.37 ± 0.59	98.79 ± 0.78	50.71 ± 0.92	27.80 ± 0.62
20% Ethanol	59.62 ± 0.62	87.12 ± 1.21	36.61 ± 0.63	9.45 ± 0.19
10% Ethanol	59.46 ± 0.48	60.11 ± 0.82	19.28 ± 0.31	0.88 ± 0.21
0% Ethanol	43.75 ± 0.49	42.56 ± 0.59	3.32 ± 0.29	0.00 ± 0.00

* Significant differences between solvents (*p* < 0.05) were determined using a one-way ANOVA.

## Data Availability

The original contributions presented in this study are included in the article. Further inquiries can be directed to the corresponding author.

## Supplementary Materials

The following supporting information can be downloaded at https://www.mdpi.com/article/10.3390/molecules31203586/s1, Figure S1: Chemical structures of oroxin A, oroxin B, baicalein and chrysin in *O. indicum*; Figure S2: Raw materials from different organs of *O. indicum*; Table S1: Physicochemical properties of oroxin B, oroxin A, baicalein, and chrysin calculated using SwissADME.

## Abbreviations

The following abbreviations are used in this manuscript:

AOAC	Association of Official Analytical Collaboration
C18	Octadecylsilane
°C	Celsius degree
cm	Centimeter
EtOH	Ethanol
HPLC	High-Performance Liquid Chromatography
HSP	Hansen solubility parameter
ICH	The International Council for Harmonisation of Technical Requirements for Pharmaceuticals for Human Use
LOD	Limit of Detection
LOQ	Limit of Quantitation
mL	Milliliter
Mol	Mole
MS	Mass spectrometry
PDA	Photo Diode-Array
R^2^	Coefficient of determination
Ra	Hansen solubility parameter distance
RT	Retention time
SD	Standard deviation
UHPLC	Altra High-Performance Liquid Chromatography
UV	Ultraviolet
δD	Dispersion force
δH	Hydrogen bonding energy
δP	Dipolar intermolecular force
µg	Micro gram
%RSD	Percentage relative standard deviation
λmax	Wavelength of Maximum Absorbance
