# Supplementary material for: Application of Hansen Solubility Parameters for Green Solvent Extraction of Flavonoids from Oroxylum indicum (L.) Kurz and Chromatographic Profiling Across Plant Organs
Source: Molecules. 2026 Oct 9;31(20):3586. doi: 10.3390/molecules31203586 (PMC13707399; doi:10.3390/molecules31203586)
Supplement: Supplementary file 1 [file molecules-31-03586-s001.zip › molecules-4566212-supplementary.pdf]

Supplementary Information

1. Physicochemical properties of baicalein derivatives

The four major flavonoids can be classified by chemical structure into two groups: flavonoid glycosides (oroxin B and oroxin A) and flavonoid aglycones (baicalein and chrysin). Oroxin B is a disaccharide glycoside of baicalein, whereas oroxin A is a monosaccharide glycoside; chrysin is a 6-dehydroxy derivative of baicalein (Figure S1). These structural differences result in distinct physicochemical properties: calculated logP and total polar surface area (TPSA) values obtained from SwissADME reflect differences in aqueous solubility among the baicalein derivatives. As reported in Table S1, the predicted water solubility decreases in the order: oroxin B > oroxin A > chrysin > baicalein.

Table S1. Physicochemical properties of oroxin B, oroxin A, baicalein, and chrysin calculated using SwissADME.

Properties	Oroxin B	Oroxin A	Baicalein	Chrysin
Molecular Weight (g/mole)	594.52	432.38	270.24	254.24
Log $P_{o/w}$	−1.20	0.41	2.24	2.02
TPSA (Å^2)	249.20	170.05	90.90	70.67
Water solubility (mg/mL)	4.33 x 10^{-1}	2.85 x 10^{-1}	2.51 x 10^{-2}	1.06 x 10^{-1}

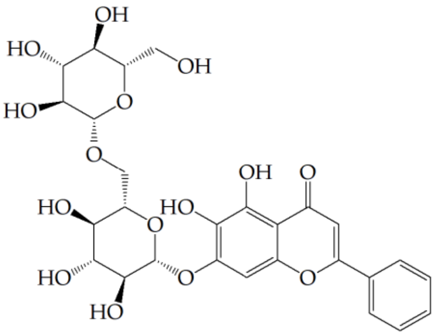

Oroxin B

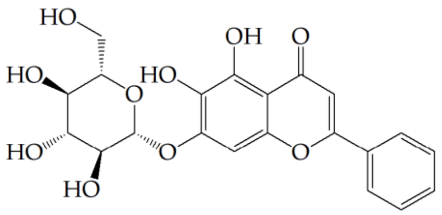

Oroxin A

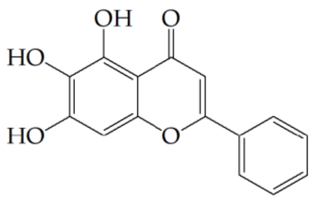

Baicalein

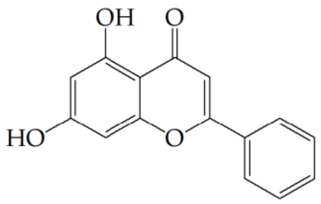

Chrysin

Figure S1. Chemical structures of oroxin A, oroxin B, baicalein and chrysin in *O. indicum***.**

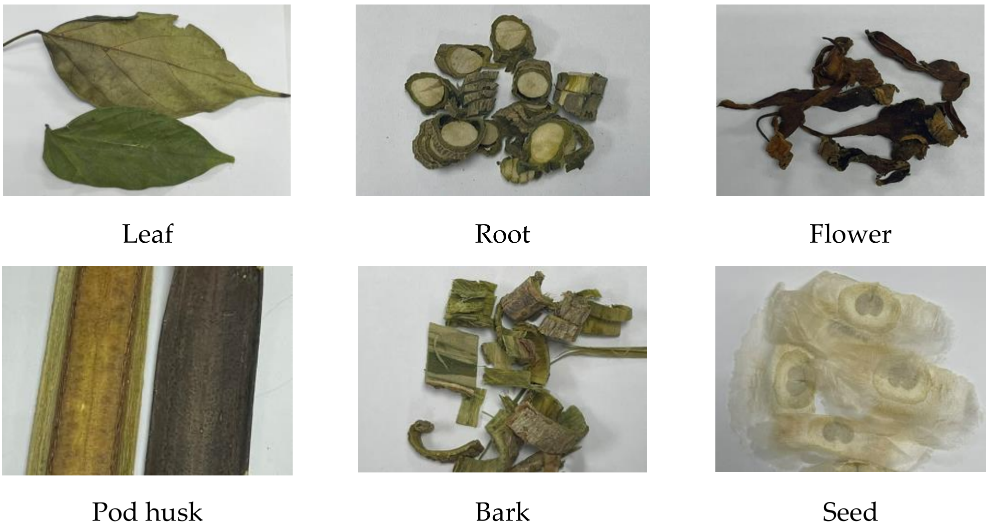

Figure S2. Raw materials from different organs of *O. indicum*.
